# Di-μ-chlorido-bis­[chlorido(*N*,*N*-di­methyl­ethylenediamine-κ^2^ *N*,*N*′)zinc(II)]

**DOI:** 10.1107/S1600536809019473

**Published:** 2009-06-06

**Authors:** Ming-Ming Yu, Qiu-Zhi Shi, Yu-Na Zhang, Zhan-Xian Li

**Affiliations:** a Department of Chemistry, Zhengzhou University, Zhengzhou 450001, People’s Republic of China

## Abstract

The centrosymmetric dinuclear title compound, [Zn_2_Cl_4_(C_4_H_12_N_2_)_2_], is isostructural with its previously reported Cu^II^ analogue [Phelps, Goodman & Hodgson (1976 ▶). *Inorg. Chem.* **15**, 2266–2270]. In the title compound, each of the Zn^II^ ions is coordinated by two N atoms from a chelating *N*,*N*-dimethyl­ethylenediamine ligand, two bridging Cl atoms and one terminal Cl atom. The coordination environment is distorted square-pyramidal. The Zn—Cl bond distances of the two bridging Cl atoms are distinctly different: the equatorial Cl atom exbibits a Zn—Cl distance of 2.318 (1) Å and the axial Cl atom exbibits a Zn—Cl distance of 2.747 (2) Å, which is significantly longer. The mol­ecule can thus be seen as a dimer of two nearly square-planar monomeric units which are related to each other by an inversion center located in the middle of the dimer. Within one monomeric unit, the Zn atom, the two N atoms and the two Cl atoms are almost coplanar, with a mean deviation of only 0.05 (1) Å from the associated least-squares plane. The Zn⋯Zn distance within the dimer is 3.472 (3) Å. N—H⋯Cl and C—H⋯Cl hydrogen-bond inter­actions connect neighboring mol­ecules with each other.

## Related literature

For the isostructural Cu^II^ complex, see: Phelps *et al.* (1976 ▶). For general background on the coordination behaviour of *N*,*N*-dimethyl­ethylenediamine, see: Basak *et al.* (2007 ▶); Hlavinka & Hagadorn (2003 ▶); Knight *et al.* (2008 ▶). Allen (2002 ▶) describes the Cambridge Structural Database.
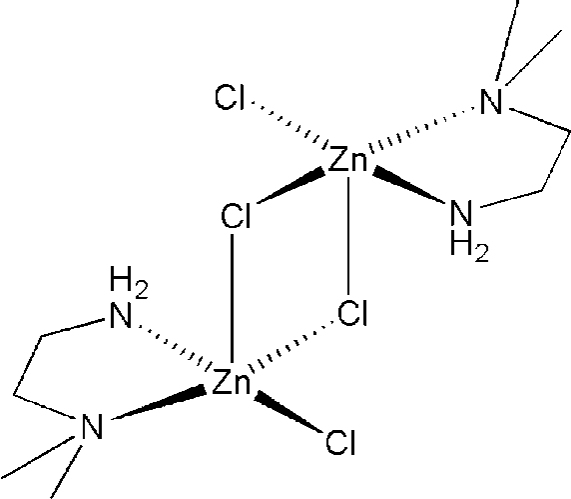


## Experimental

### 

#### Crystal data

[Zn_2_Cl_4_(C_4_H_12_N_2_)_2_]
*M* *_r_* = 448.85
Orthorhombic, 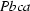

*a* = 9.808 (2) Å
*b* = 8.5109 (17) Å
*c* = 20.851 (4) Å
*V* = 1740.5 (6) Å^3^
*Z* = 4
Mo *K*α radiation
μ = 3.36 mm^−1^
*T* = 295 K
0.15 × 0.12 × 0.07 mm

#### Data collection

Bruker SMART 1K CCD area-detector diffractometer
Absorption correction: multi-scan (*SADABS*; Bruker, 2000 ▶) *T*_min_ = 0.633, *T*_max_ = 0.799
7050 measured reflections
1620 independent reflections
1300 reflections with *I* > 2σ(*I*)
*R*_int_ = 0.042

#### Refinement

*R*[*F* ^2^ > 2σ(*F* ^2^)] = 0.049
*wR*(*F* ^2^) = 0.132
*S* = 1.10
1620 reflections
82 parameters
H-atom parameters constrained
Δρ_max_ = 1.14 e Å^−3^
Δρ_min_ = −0.42 e Å^−3^

### 

Data collection: *SMART* (Bruker, 2000 ▶); cell refinement: *SAINT* (Bruker, 2000 ▶); data reduction: *SAINT*; program(s) used to solve structure: *SHELXTL* (Sheldrick, 2008 ▶); program(s) used to refine structure: *SHELXTL*; molecular graphics: *SHELXTL*; software used to prepare material for publication: *SHELXTL*.

## Supplementary Material

Crystal structure: contains datablocks I, global. DOI: 10.1107/S1600536809019473/zl2193sup1.cif

Structure factors: contains datablocks I. DOI: 10.1107/S1600536809019473/zl2193Isup2.hkl

Additional supplementary materials:  crystallographic information; 3D view; checkCIF report

## Figures and Tables

**Table 1:** Hydrogen-bond geometry (Å, °)

*D*—H⋯*A*	*D*—H	H⋯*A*	*D*⋯*A*	*D*—H⋯*A*
N1—H1*D*⋯Cl1^i^	0.90	2.51	3.342 (2)	155
C4—H4*C*⋯Cl2	0.96	2.78	3.350 (9)	119
N1—H1*A*⋯Cl2^ii^	0.90	2.90	3.697 (2)	149

Symmetry codes: (i) 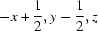; (ii) 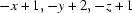.

**Fig. 1.**
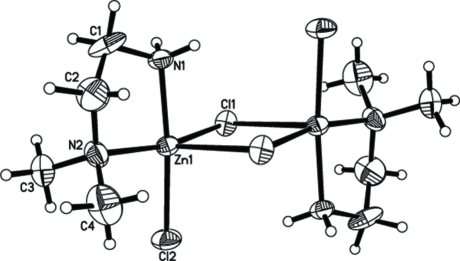
A view of title complex, showing 30% probability displacement ellipsoids and the atom-numbering scheme.

**Fig. 2.**
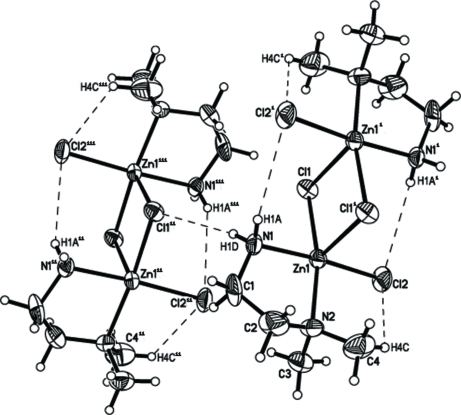
N—H···Cl and C—H···Cl interactions (dashed lines) in the title compound. [Symmetry codes: (i) 1 - x, 2 - y, 1 - z; (ii) 0.5 - x, 0.5 + y, z; (iii) 0.5 + x, 1.5 - y, 1 - z]

## supplementary crystallographic information

### Comment

*N*,*N*-Dimethylethylenediamine has the potential to function as a bidentatate nitrogen ligand by coordinating to metal ions in a chelating fashion (Hlavinka & Hagadorn, 2003; Knight *et al.*, 2008; Basak *et al.*, 2007). Here, we report the crystal structure of the title compound, an asymmetrically chloro-bridged dimeric zinc(II) complex.

In the centrosymmetric dinuclear title compound, [Zn_2_Cl_4_(C_4_H_12_N_2_)_2_], each of the Zn^II^ ions is coordinated by two N atoms from a chelating *N*,*N*-dimethylethylenediamine ligand, two bridging Cl atoms and one terminal Cl atom. The coordination environment is distorted square-pyramidal. In the dimeric structure, two Zn^II^ ions are bridged through the Cl atoms, resulting in a planar Zn_2_Cl_2_ core. The Zn—Cl bond distances of the two bridging Cl atoms are distinctly different: The equatorial Cl atoms exbibit a Zn—Cl distance of 2.318 (1) Å, the Zn—Cl distances of the axial chlorides are with 2.747 (2) Å significantely longer. The title compound could thus be considered as a dimer of two nearly square planar monomeric units which are related to each other by an inversion center located in the middle of the molecule [symmetry code: 1 - *x*, 2 - *y*, 1 - *z*]. Within one monomeric unit the atoms Zn1, N1, N2, Cl1 and Cl2 are almost coplanar with a mean deviation of only 0.05 (1) Å from the associated least-squares plane.

The methyl substituted N atom N2 is located opposite of the bridging Cl atom Cl1, probably due to its larger steric demand when compared to the unsubstituted NH_2_ group and due the ability to form an intramolecular N—H···Cl hydrogen bond to the terminal Cl atom in the other half of the dimer (see Table 1 and below).

The Cambridge Structural Database (Allen, 2002) does not list any crystal structures with a Zn^II^ ion in a square-pyramidal environment with two bridging Cl atoms and one terminal Cl atom. This motif seems to be more typical for Cu^II^ complexes for which the CSD has 15 entries. The structure of the title complex is indeed isostructural to its copper(II) analogue [CuCl_2_(C_4_H_12_N_2_)]_2_ (Phelps *et al.*, 1976). Both structures are very similiar, as proved by the distance of M—Cl, the M···M separation and the bridging M—Cl—M angle (Zn—Cl = 2.318 (1) Å, Zn—Cl' = 2.747 (2) Å, Zn···Zn = 3.472 (3) Å, Zn—Cl—Zn = 86.11 (4) °; Cu—Cl = 2.309 (2) Å, Cu—Cl' = 2.734 (3) Å, Cu···Cu = 3.458 (3) Å, Cu—Cl—Cu = 86.11 (8) °.

In the crystal structure, the dimer is strengthened by intramolecular hydrogen bond interactions involving the methyl and amino protons of the ligand and the terminal Cl atom [C4—H4C···Cl2 and N1—H1A···Cl2^i^, symmetry code: (i) 1 - *x*, 2 - *y*, 1 - *z*]. An intermolecular N1—H1D···Cl1^ii^ hydrogen bond interaction between the other amino H atom and one of the bridging Cl atoms leads to the formation of a one-dimensional supramolecular chain (Table 1, Fig. 2).

### Experimental

Colourless crystals of the title complex were obtained by slow evaporation of a solution in ethanol (20 ml) and water (5 ml) of *N*,*N*-dimethylethylenediamine (0.044 g, 0.5 mmol) and ZnCl_2_ (0.068 g, 0.5 mmol). Yield, 85%. Selected IR data (cm^-1^, KBr pellet): 3342, 3285 (*m*), 3161 (w), 3048 (w), 1465 (*m*), 1332 (w), 1292 (w), 1248 (w), 1189 (w), 1007 (*m*), 937 (w), 896 (w), 789(w), 631 (*m*). Anal. Calcd for C_8_H_24_Cl_4_N_4_Zn_2_ requires C, 21.4; H, 5.39; N, 12.48. Found: C, 21.1; H, 5.41; N, 12.23%.

### Refinement

The H atoms bound to C and N atoms were placed in caculated positions with C—H = 0.97 Å (CH_2_), C—H = 0.96 Å (CH_3_) and with *U*_iso_(H) = 1.2*U*_eq_(C), and with N—H distances of 0.90 Å and with *U*_iso_(H) = 1.2*U*_eq_(N).

### Crystal data

**Table:**

[Zn_2_Cl_4_(C_4_H_12_N_2_)_2_]	*F*(000) = 912
*M**_r_* = 448.85	*D*_x_ = 1.713 Mg m^−^^3^
Orthorhombic, *P**b**c**a*	Mo *K*α radiation, λ = 0.71073 Å
Hall symbol: -P 2ac 2ab	Cell parameters from 1620 reflections
*a* = 9.808 (2) Å	θ = 2.0–25.5°
*b* = 8.5109 (17) Å	µ = 3.36 mm^−^^1^
*c* = 20.851 (4) Å	*T* = 295 K
*V* = 1740.5 (6) Å^3^	Block, colourless
*Z* = 4	0.15 × 0.12 × 0.07 mm

### Data collection

**Table:**

Bruker SMART 1K CCD area-detector diffractometer	1620 independent reflections
Radiation source: fine-focus sealed tube	1300 reflections with *I* > 2σ(*I*)
graphite	*R*_int_ = 0.042
φ and ω scans	θ_max_ = 25.5°, θ_min_ = 2.0°
Absorption correction: multi-scan (*SADABS*; Bruker, 2000)	*h* = −11→11
*T*_min_ = 0.633, *T*_max_ = 0.799	*k* = −6→10
7050 measured reflections	*l* = −25→20

### Refinement

**Table:**

Refinement on *F*^2^	Primary atom site location: structure-invariant direct methods
Least-squares matrix: full	Secondary atom site location: difference Fourier map
*R*[*F*^2^ > 2σ(*F*^2^)] = 0.049	Hydrogen site location: inferred from neighbouring sites
*wR*(*F*^2^) = 0.132	H-atom parameters constrained
*S* = 1.10	*w* = 1/[σ^2^(*F*_o_^2^) + (0.0632*P*)^2^ + 2.354*P*] where *P* = (*F*_o_^2^ + 2*F*_c_^2^)/3
1620 reflections	(Δ/σ)_max_ < 0.001
82 parameters	Δρ_max_ = 1.14 e Å^−^^3^
0 restraints	Δρ_min_ = −0.41 e Å^−^^3^

### Special details

**Table:**

Geometry. All e.s.d.'s (except the e.s.d. in the dihedral angle between two l.s. planes) are estimated using the full covariance matrix. The cell e.s.d.'s are taken into account individually in the estimation of e.s.d.'s in distances, angles and torsion angles; correlations between e.s.d.'s in cell parameters are only used when they are defined by crystal symmetry. An approximate (isotropic) treatment of cell e.s.d.'s is used for estimating e.s.d.'s involving l.s. planes.
Refinement. Refinement of *F*^2^ against ALL reflections. The weighted *R*-factor *wR* and goodness of fit *S* are based on *F*^2^, conventional *R*-factors *R* are based on *F*, with *F* set to zero for negative *F*^2^. The threshold expression of *F*^2^ > σ(*F*^2^) is used only for calculating *R*-factors(gt) *etc*. and is not relevant to the choice of reflections for refinement. *R*-factors based on *F*^2^ are statistically about twice as large as those based on *F*, and *R*- factors based on ALL data will be even larger.

### Fractional atomic coordinates and isotropic or equivalent isotropic displacement parameters (Å^2^)

**Table:**

	*x*	*y*	*z*	*U*_iso_*/*U*_eq_	
Zn1	0.43590 (6)	0.94329 (7)	0.42593 (3)	0.0433 (3)	
Cl1	0.31460 (12)	1.00877 (18)	0.51751 (7)	0.0526 (4)	
Cl2	0.40919 (18)	1.18349 (18)	0.38224 (8)	0.0700 (5)	
N1	0.4424 (4)	0.7235 (5)	0.4593 (2)	0.0490 (11)	
H1A	0.5032	0.7173	0.4915	0.059*	
H1D	0.3600	0.6964	0.4748	0.059*	
N2	0.5142 (5)	0.8454 (6)	0.3424 (2)	0.0566 (12)	
C3	0.4065 (7)	0.8464 (9)	0.2933 (3)	0.077 (2)	
H3A	0.4408	0.8009	0.2544	0.116*	
H3B	0.3784	0.9527	0.2853	0.116*	
H3C	0.3299	0.7864	0.3082	0.116*	
C1	0.4819 (7)	0.6142 (7)	0.4073 (4)	0.078 (2)	
H1B	0.5299	0.5250	0.4254	0.093*	
H1C	0.4005	0.5752	0.3861	0.093*	
C4	0.6311 (8)	0.9331 (11)	0.3154 (4)	0.099 (3)	
H4A	0.6624	0.8815	0.2772	0.148*	
H4B	0.7036	0.9364	0.3463	0.148*	
H4C	0.6031	1.0382	0.3051	0.148*	
C2	0.5679 (9)	0.6908 (9)	0.3608 (4)	0.095 (3)	
H2A	0.5753	0.6251	0.3229	0.114*	
H2B	0.6586	0.7035	0.3787	0.114*	

### Atomic displacement parameters (Å^2^)

**Table:**

	*U*^11^	*U*^22^	*U*^33^	*U*^12^	*U*^13^	*U*^23^
Zn1	0.0489 (4)	0.0326 (4)	0.0485 (4)	0.0042 (2)	−0.0006 (3)	0.0006 (2)
Cl1	0.0422 (6)	0.0581 (8)	0.0575 (8)	0.0054 (6)	−0.0002 (6)	−0.0108 (6)
Cl2	0.0875 (11)	0.0378 (8)	0.0847 (11)	0.0070 (7)	−0.0056 (9)	0.0152 (7)
N1	0.056 (3)	0.035 (2)	0.056 (3)	−0.0057 (19)	0.000 (2)	0.0071 (19)
N2	0.074 (3)	0.057 (3)	0.039 (2)	0.014 (2)	0.000 (2)	−0.002 (2)
C3	0.089 (5)	0.086 (5)	0.058 (4)	−0.007 (4)	−0.015 (3)	−0.011 (4)
C1	0.074 (4)	0.026 (3)	0.133 (6)	−0.002 (3)	0.026 (4)	−0.004 (3)
C4	0.071 (5)	0.146 (9)	0.079 (5)	−0.003 (5)	0.024 (4)	−0.005 (5)
C2	0.133 (7)	0.071 (5)	0.080 (5)	0.037 (5)	0.015 (5)	−0.016 (4)

### Geometric parameters (Å, °)

**Table:**

Zn1—N1	1.997 (4)	C3—H3A	0.9600
Zn1—N2	2.078 (4)	C3—H3B	0.9600
Zn1—Cl2	2.2533 (16)	C3—H3C	0.9600
Zn1—Cl1	2.3179 (14)	C1—C2	1.441 (10)
Zn1—Cl1^i^	2.7468 (15)	C1—H1B	0.9700
Cl1—Zn1^i^	2.7468 (15)	C1—H1C	0.9700
N1—C1	1.481 (8)	C4—H4A	0.9600
N1—H1A	0.9000	C4—H4B	0.9600
N1—H1D	0.9000	C4—H4C	0.9600
N2—C2	1.468 (9)	C2—H2A	0.9700
N2—C3	1.471 (8)	C2—H2B	0.9700
N2—C4	1.480 (9)		
			
N1—Zn1—N2	84.52 (19)	N2—C3—H3B	109.5
N1—Zn1—Cl2	173.95 (13)	H3A—C3—H3B	109.5
N2—Zn1—Cl2	93.89 (14)	N2—C3—H3C	109.5
N1—Zn1—Cl1	87.40 (14)	H3A—C3—H3C	109.5
N2—Zn1—Cl1	167.59 (16)	H3B—C3—H3C	109.5
Cl2—Zn1—Cl1	93.17 (6)	C2—C1—N1	111.2 (5)
N1—Zn1—Cl1^i^	87.78 (13)	C2—C1—H1B	109.4
N2—Zn1—Cl1^i^	95.19 (14)	N1—C1—H1B	109.4
Cl2—Zn1—Cl1^i^	98.19 (6)	C2—C1—H1C	109.4
Cl1—Zn1—Cl1^i^	93.89 (4)	N1—C1—H1C	109.4
Zn1—Cl1—Zn1^i^	86.11 (4)	H1B—C1—H1C	108.0
C1—N1—Zn1	110.0 (4)	N2—C4—H4A	109.5
C1—N1—H1A	109.7	N2—C4—H4B	109.5
Zn1—N1—H1A	109.7	H4A—C4—H4B	109.5
C1—N1—H1D	109.7	N2—C4—H4C	109.5
Zn1—N1—H1D	109.7	H4A—C4—H4C	109.5
H1A—N1—H1D	108.2	H4B—C4—H4C	109.5
C2—N2—C3	116.5 (6)	C1—C2—N2	111.8 (6)
C2—N2—C4	105.9 (6)	C1—C2—H2A	109.3
C3—N2—C4	106.8 (5)	N2—C2—H2A	109.3
C2—N2—Zn1	105.8 (4)	C1—C2—H2B	109.3
C3—N2—Zn1	108.4 (4)	N2—C2—H2B	109.3
C4—N2—Zn1	113.8 (4)	H2A—C2—H2B	107.9
N2—C3—H3A	109.5		
			
N1—Zn1—Cl1—Zn1^i^	87.59 (12)	Cl2—Zn1—N2—C3	−65.5 (4)
N2—Zn1—Cl1—Zn1^i^	136.9 (6)	Cl1—Zn1—N2—C3	59.0 (8)
Cl2—Zn1—Cl1—Zn1^i^	−98.44 (6)	Cl1^i^—Zn1—N2—C3	−164.1 (4)
Cl1^i^—Zn1—Cl1—Zn1^i^	0.0	N1—Zn1—N2—C4	−132.8 (5)
N2—Zn1—N1—C1	−6.1 (4)	Cl2—Zn1—N2—C4	53.0 (5)
Cl1—Zn1—N1—C1	164.4 (4)	Cl1—Zn1—N2—C4	177.6 (5)
Cl1^i^—Zn1—N1—C1	−101.6 (4)	Cl1^i^—Zn1—N2—C4	−45.6 (5)
N1—Zn1—N2—C2	−17.0 (5)	Zn1—N1—C1—C2	29.5 (7)
Cl2—Zn1—N2—C2	168.8 (5)	N1—C1—C2—N2	−46.2 (9)
Cl1—Zn1—N2—C2	−66.6 (8)	C3—N2—C2—C1	−82.0 (8)
Cl1^i^—Zn1—N2—C2	70.2 (5)	C4—N2—C2—C1	159.5 (7)
N1—Zn1—N2—C3	108.6 (4)	Zn1—N2—C2—C1	38.5 (8)

Symmetry codes: (i) −*x*+1, −*y*+2, −*z*+1.

### Hydrogen-bond geometry (Å, °)

**Table:**

*D*—H···*A*	*D*—H	H···*A*	*D*···*A*	*D*—H···*A*
N1—H1D···Cl1^ii^	0.90	2.51	3.342 (2)	155
C4—H4C···Cl2	0.96	2.78	3.350 (9)	119
N1—H1A···Cl2^i^	0.90	2.90	3.697 (2)	149

Symmetry codes: (ii) −*x*+1/2, *y*−1/2, *z*; (i) −*x*+1, −*y*+2, −*z*+1.
